# Hemophagocytic Lymphohistiocytosis in a Patient With Post-acute COVID-19 Infection

**DOI:** 10.7759/cureus.31451

**Published:** 2022-11-13

**Authors:** Mohamed Zakee Mohamed Jiffry, Mohammed Ahmed-khan, Jonathan Vargas, Teena Thomas, Susanna Josey

**Affiliations:** 1 Internal Medicine, Danbury Hospital, Danbury, USA

**Keywords:** post acute sars-cov-2, hematology, autoinflammatory syndrome, h-score, covid-19, secondary hemophagocytic lymphohistiocytosis (shlh), hemophagocytic lymphohistiocytosis (hlh)

## Abstract

Hemophagocytic lymphohistiocytosis (HLH) is characterized by immune dysregulation with extensive inflammation and tissue destruction due to abnormal immune activation. Post-COVID-19 patients who have recovered with negative serologic tests may also present with secondary HLH, an unusual finding that our case demonstrates.

A 73-year-old male with a notable past medical history of fall COVID-19 infection approximately 11 months prior presented initially to emergency services with a chief complaint of high fevers, lethargy, and progressive changes in mentation gradually progressive over the last 5 months’ duration. This presentation was concerning for HLH in view of the patient's high H score and clinical suspicion for HLH, and he was initiated on dexamethasone 20 mg daily. intravenous immune globulin (IVIG) protocol was also trialed, despite which the patient continued to deteriorate before expiring during the course of the hospitalization.

sHLH following COVID-19 infection remains a poorly understood phenomenon. The severity of the COVID-19 infection does not appear to be related to one's predisposition to develop sHLH. The mortality of HLH remains high even with appropriate therapy.

## Introduction

Hemophagocytic lymphohistiocytosis (HLH) is a syndrome characterized by abnormal immune activation. It is thought to be due to hyperinflammation concomitant with immune dysregulation with failure of innate mechanisms to restrict abnormal activation of macrophages by natural killers (NKs) and/or cytotoxic T lymphocytes (CTLs) [1].

HLH is commonly subtyped as primary or secondary (sHLH), with the understanding that primary HLH arises secondary to genetic mutations, while secondary HLH is associated with infections, malignancy, immunodeficiency, or rheumatologic syndromes, although this distinction between primary and sHLH may cause confusion as both can be triggered by infections or other immune activating events [2].

Although HLH in COVID-19-positive patients is a recognized entity, post-COVID-19 patients who have recovered and are negative serologically may also be at increased risk for HLH as our case demonstrates, although this is a rare phenomenon and is poorly described by existing literature [3].

## Case presentation

A 73-year-old male presented initially to emergency services for further evaluation of persistent fevers for the past 5 months. This was accompanied by progressive lethargy and fluctuations in his level of alertness. The patient stated that his fevers were persistent, recorded up to 105°F.

Past medical history for the patient was significant for a history of COVID-19 infection 11 months prior to his current presentation which was complicated by pneumonia for which he required hospitalization, and from which he completely recovered following treatment. His other medical conditions include a history of benign prostatic hyperplasia for which he had undergone transurethral resection, iron deficiency anemia, and sleep apnea. He had a recent hospitalization of 5 days duration, 2 weeks prior to this presentation at a neighboring hospital for similar complaints of persistent fevers with lethargy. At the time, a bone marrow biopsy and aspirate were done which revealed no evidence of leukemia/lymphoma, myeloid dysplasia, or hemophagocytosis.

The patient has had no prior travel history of particular note, nor does he have any family history of hematologic malignancy or immunodeficiency syndromes. Physical examination showed a lethargic and confused male. Extremities were warm to the touch. Otherwise, no rashes or petechiae were noted. The cardiovascular and respiratory exam was unremarkable. His abdomen was soft and nontender with no organomegaly appreciated.

Initial lab work included a complete blood count which showed normocytic anemia and thrombocytopenia. White blood cell count was normal initially but downtrend over the course of his hospitalization with a low absolute neutrophil count as well. Liver enzyme derangement was present, and notably, the aspartate transaminase (AST): alanine transaminase (ALT) ratio was 2:1. Lactate dehydrogenase (LDH), ferritin, and triglycerides were elevated. Coagulation parameters were deranged with a high international normalized ratio (INR), prothrombin time (PT), and activated partial thromboplastin time (APTT). D-dimer was elevated and low fibrinogen levels were also noted (Table 1).

**Table 1:** Laboratory investigations on days 1 and 3 of hospitalization. Laboratory investigations through the course of the hospitalization at hospital days 1 and 3 post-admission are presented. Investigations are tabulated with lab-specific reference ranges. WBC: white blood cell count; ANC: absolute neutrophil count; AST: aspartate transaminase; ALT: alanine transaminase; PT: prothrombin time; APTT: activated partial thromboplastin time; LDH: lactate dehydrogenase, INR: international normalized ratio

	Day since admission		
Laboratory investigation (units)	Day 1	Day 3	Reference range
Complete blood count:			
Hemoglobin (g/dL)	7.8	6.8	13.5-17.0
Platelets (10^9/L)	13	<10	150-400
WBC (10^9/L)	5.5	0.4	3.5-10
ANC (10^9/L)	0.66	-	2.0-7.5
Liver function tests:			
AST (units/L)	153	86	10-50
ALT (units/L)	73	59	10-62
Alkaline phosphatase (units/L)	169	101	40-130
Coagulation studies:			
PT (seconds)	24.1	19.7	12.2-14.5
INR	2.21	1.71	0.91-1.40
APTT (seconds)	51.9	36.6	25.0-35.8
D-dimer (ng/mL)	3780	8,960	<= 500
Fibrinogen (mg/dL)	38	36.6	210-480
LDH (units/L)	725	374	122-222
Ferritin (ng/mL)	60,435	42,002	24-370
Triglycerides (mg/dL)	268	-	<= 149

A peripheral blood smear was obtained which showed leukocytopenia with absolute neutropenia and lymphocytopenia. A mild neutrophilic left shift was noted with lymphocytes showing reactive features. Critical thrombocytopenia with normocytic normochromic anemia was noted but circulating blasts, platelet clumps, or schistocytes were not seen. Severe acute respiratory syndrome coronavirus 2 (SARS-CoV-2) RNA testing was negative during this hospitalization. Blood group and cross-match showed an O-negative ABO/Rh status.

**Figure 1:**
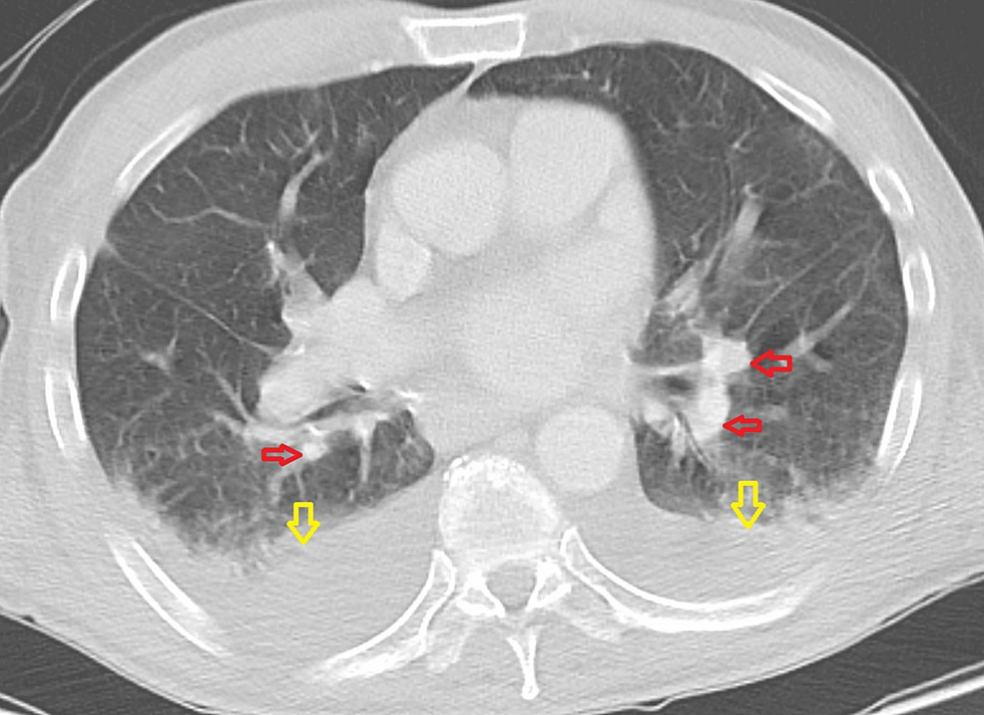
Computed tomography (CT) scan of the chest on admission. Red arrows: Scattered focal and ground-glass opacities noted peripherally in both lung fields. Yellow arrows: Bilateral pleural effusions with areas of compressive atelectasis/infiltrate posteriorly.

**Figure 2:**
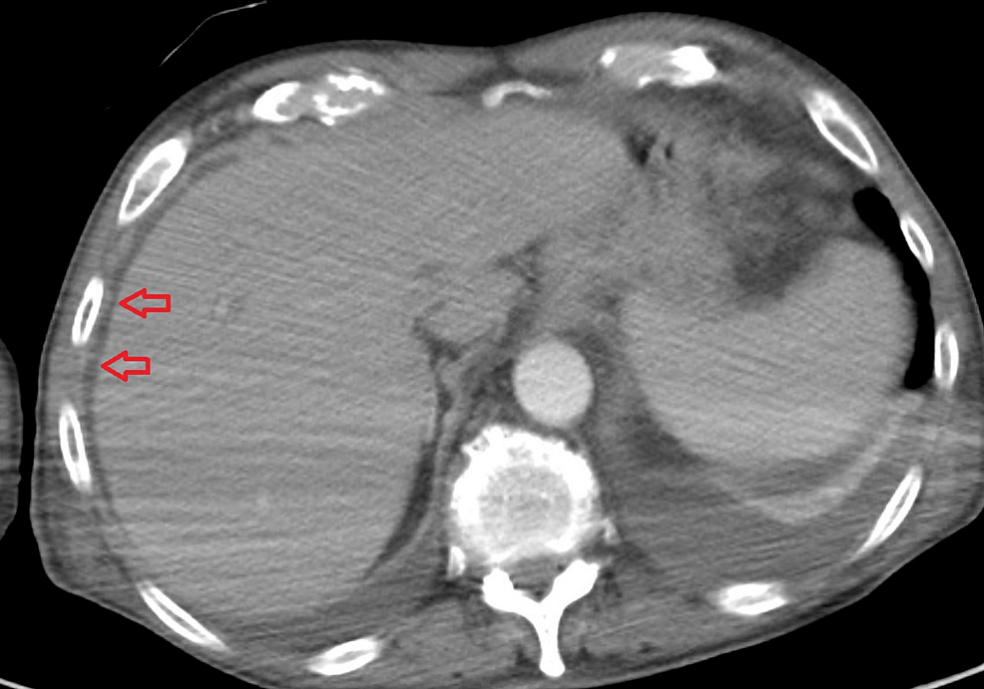
Computed tomography (CT) scan of the abdomen on admission. Red arrows: Area of mild perihepatic fluid collection noted.

Imaging investigations included a CT chest abdomen pelvis with contrast which showed bilateral pleural effusions with compressive atelectasis/infiltrate. Multiple small peripheral opacities in the left upper lobe, right upper lobe, and right middle lobe were noted (Figure 1). Mild perihepatic fluid and free fluid in the pelvis consistent with mild ascites were noted (Figure 2). CT brain was unremarkable for evidence of intracranial pathology.

An H score for the reactive hemophagocytic syndrome was subsequently calculated. The patient scored 0 points for known underlying immunosuppression, 33 points for temperature recorded at greater than 102.9oF, 0 points for no organomegaly, 34 points for trilineage cytopenia, 50 points for ferritin greater than 6000 ng/mL, 44.4 elevated triglyceride between 132.7 and 354, 30 points for fibrinogen level less than 250 mg/dL, 19 points for an AST greater than 30 and 0 points for features of hemophagocytosis on bone marrow aspirate. The total score was 226 points which places the patient at 96 to 98% probability of hemophagocytic syndrome.

Following admission and in view of the high likelihood of HLH, the patient was started on high-dose steroid therapy with dexamethasone 20 mg daily. However, the patient’s clinical status continued to deteriorate over the subsequent days. Worsening leukopenia on the third day of hospitalization with critical neutropenia prompted the initiation of filgrastim 480 mcg daily. Transfusional support with blood products for severe thrombocytopenia and deranged coagulation parameters included a total of six packs of cytomegalovirus (CMV)-negative irradiated leukocyte reduced platelets, four units of fresh frozen plasma, six units of cryoprecipitate, and three units of leukocyte reduced packed red blood cells. Although suspicion for an infective process was low, 4 days of antibiotic therapy with cefepime 2 g q8 and vancomycin dosed per pharmacy protocol was administered. Symptomatic relief of pain was achieved with acetaminophen.

On the fourth day of hospitalization, intravenous immunoglobulin therapy was started. The patient was continued on dexamethasone 20 mg daily. The patient continued to deteriorate despite 3 days of intravenous immunoglobulin therapy, and a palliative care discussion was held with the family, following which goals of care were transitioned to comfort measures only, and all active treatment was stopped which included intravenous immune globulin (IVIG), steroids, and antibiotics. Morphine for symptomatic relief of pain was added to his regimen. The patient subsequently died the following day.

## Discussion

HLH is a perplexing syndrome with the principal cell types involved being macrophages, NKs, and CTLs [4]. Lack of normal feedback regulation results in excessive macrophage activation with failure of NK cells and CTLs to eliminate the activated macrophages [5].

Another characteristic feature of the syndrome is hemophagocytosis which refers to the engulfment of host blood cells by macrophages. This alone is neither pathognomonic nor required for the diagnosis, and our patient did not have evidence of hemophagocytosis in the bone marrow aspirate done during his recent past hospitalization prior to presentation to our service [6]. The persistent activation of macrophages, NK cells, and CTLs also leads to excessive cytokine production with a resulting cytokine storm, and it is this that is thought to be responsible for the multiorgan failure and high mortality of the syndrome [7].

sHLH has been identified as having a broad category of triggers including those that cause immune activation and those that lead to immune deficiency. Infection is a common trigger leading to immune activation with the most common infectious triggers being viral infections, especially the Epstein-Barr virus [8]. Other viral infections that have been implicated include CMV, parvovirus, herpes simplex virus, varicella-zoster virus, measles virus, human herpes virus 8, H1N1 influenza virus, Parechovirus, and HIV [9-12].

HLH in patients with COVID-19 infection is an increasingly well-recognized phenomenon, with speculation being that the abnormal hyperinflammatory state in COVID-19 patients may induce sHLH [13,14]. Risk factors for progression to sHLH in COVID-19-infected patients may include elevated ferritin, elevated triglycerides, and/or low platelets [15]. sHLH in post-acute COVID-19 patients, which is defined as documented infection at least 6 weeks prior with negative repeat COVID-19 PCR testing, is an exceedingly unusual finding with only a few cases having been reported in the existing literature, and it remains unclear why certain patients develop HLH after the acute phase of infection [16]. Certainly, our patient is an outlier with regards to the infectious trigger having occurred more than 10 months ago, however, no alternative trigger could be identified despite extensive investigation.

The diagnosis of HLH is suggested by a compatible clinical presentation, and a scoring system termed the “H score” has been devised to estimate the probability of HLH. An H score greater than or equal to 250 confers a 99% probability of HLH, wears a score of less than 90 confers a 1% probability of the same. The H score may also have prognostic significance in addition to its diagnostic value in patients with COVID-19 and sHLH, with at least one study reporting improved mortality rates with lower H scores [17]. A markedly elevated ferritin such as our case demonstrated is also an independent predictor of increased mortality.

HLH carries a high mortality in patients without therapy, and even those patients initiated on standard therapy such as the HLH-94 protocol (which consists of high-dose dexamethasone, etoposide, and intrathecal therapy for CNS involvement) may have a poor prognosis [18]. In our case, owing to the patient’s marked clinical deterioration despite steroid therapy (including worsening liver functions indicating severe liver disease), therapy with etoposide was deferred. Intrathecal therapy was deferred in view of marked coagulopathy and thrombocytopenia despite blood product administration. Intravenous immunoglobulin has been used for HLH patients with the underlying rheumatologic disease and other causes of sHLH in adult patients, although literature surrounding its use in sHLH from COVID-19 infection is lacking. A table comparing and contrasting the characteristics of sHLH in post-acute COVID-19 and acute COVID-19 patients identified in the literature review is also presented (Table 2).

**Table 2:** Tabular comparison of characteristics of patients diagnosed with SARS-CoV-2 and sHLH identified on literature review. LMWH: low molecular weight heparin; CVVH: continuous venovenous hemodiafiltration; CRRT: continuous renal replacement therapy; IVIG: intravenous immune globulin

	Sex	Age (years)	Duration since SARS-CoV-2 infection (Acute vs post-acute)	Peak ferritin (ng/L)	H-score	Probability of sHLH per H-score (%)	Treatments received	Days to final outcome (death vs discharge)
Patient 1 ^[3]^	Female	40	60 days (post-acute)	170	213	93-96	Dexamethasone, LMWH, antibiotics, fluids	30 days (discharged)
Patient 2 ^[3]^	Male	2	14 days (acute)	188	239	98-99	Steroids, anti-epileptics, antibiotics, fluids	Not reported
Patient 3 ^[13]^	Male	70	7 days (acute)	36,023	221	96-98	Tocilizumab, antibiotics, CVVH, vasopressors	Not reported
Patient 4 ^[16]^	Male	54	42 days (post-acute)	102,000	197	80-88	methylprednisolone => dexamethasone, etoposide, vasopressors, CRRT	Not reported
Patient 5 ^[19]^	Male	20	56 days (post-acute)	21,059	174	54-70	Dexamethasone, etoposide	Not reported
Patient 6 ^[20]^	Male	40	28 days (acute)	76,225	246	>99	Anakinra, antibiotics	74 days (died)
Patient 7 ^[20]^	Male	28	6 days (acute)	3,164	182	70-80	Anakinra, antibiotics	7 days (died)
Patient 8 ^[20]^	Female	60	3 days (acute)	12,402	157	25-40	Glucocorticoids, anakinra, IVIG	43 days (died)
Patient 9 ^[20]^	Male	63	0 days (acute)	17,790	162	40-54	Anakinra, antibiotics	11 days (discharged)
Patient 10 ^[20]^	Female	22	9 days (acute)	45,864	162	40-54	Glucocorticoids, anakinra, IVIG, cyclosporine	40 days (discharged)

## Conclusions

sHLH following COVID-19 infection, although previously described, remains a poorly understood phenomenon. Even more puzzling is why certain patients develop a delayed sHLH following post-acute COVID-19 illness. Notably, the severity of the initial COVID-19 infection does not appear to be related to one’s predisposition to develop sHLH.

Regardless of etiology, the mortality of HLH remains high even with appropriate therapy, especially with neurologic involvement and markedly elevated ferritin levels. The H score remains an invaluable aid to the diagnosis of sHLH in COVID-19 patients, although further studies are warranted to ascertain a prognostic significance in these patients.
